# 

**DOI:** 10.3201/eid0804.

**Published:** 2002-04

**Figure:**
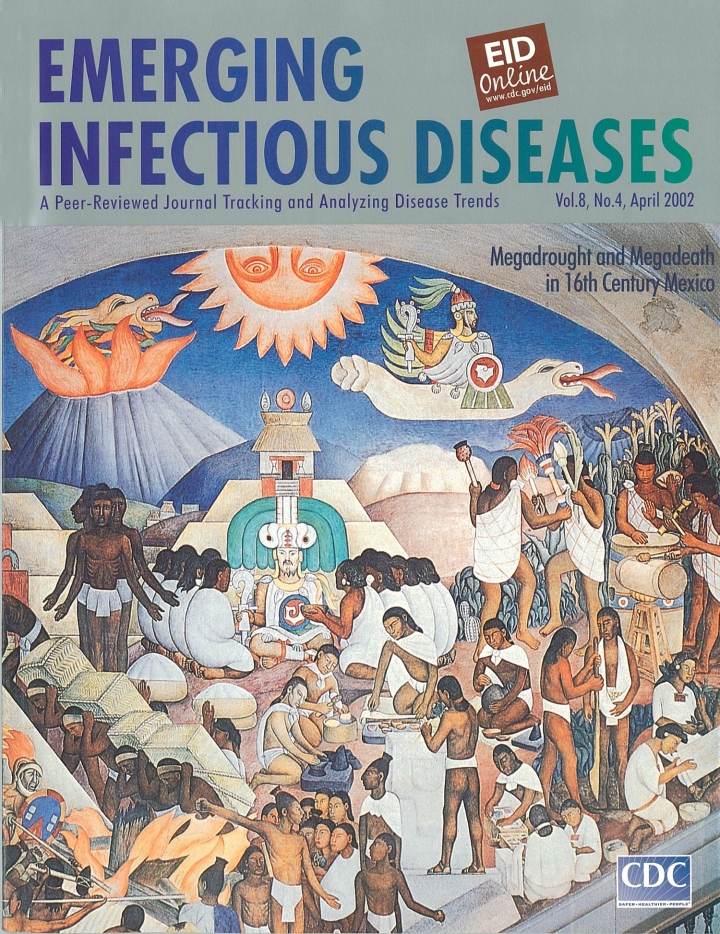
México prehispánico, El antiguo mundo indígena (Indigenous World) (1929)  Diego Rivera Fresco, North Wall National Palace, Mexico City

Printed with permission from Banco de México Diego Rivera and Frida Kahlo Museums Trust; Avenue Cinco de Mayo No. 2, Col. Centro; Del Cuauhtémoc, 06059, México.

Along with colonial conquest, infectious diseases forever changed the Aztec world in the 16th century. These diseases included smallpox and an indigenous highly lethal hemorrhagic fever known as “cocoliztli.” Tree-ring data suggest that the 16th-century cocoliztli epidemics occurred during extreme drought conditions, which may have contributed to the staggering demographic collapse of early colonial Mexico.

Until the colonial uprising of 1910, the indigenous people of Mexico had been oppressed, their individualism and Aztec origins discouraged. Diego Rivera’s mural at the National Palace in Mexico City celebrates Aztec origins, promotes a Mexican identity, and promotes the reforms of the 1910 uprising. 

The mural depicts Mexico’s mythical and precolonial past. The bright orange sun against the blue sky and the erupting volcano are life symbols of Mexican ancestors. Quetzalcoatl, mythical creator of the world, appears in three forms: star, god, and human. At first, Quetzalcoatl, who was created by serpents, sails through space as a star that accompanies the sun at night. Then, he assumes human form and comes to earth to teach the Aztec people as their king and patriarch; he sheds his blood to give them life. Finally, having completed his earthly mission, he returns to the sky. Upon leaving earth, Quetzalcoatl assumes the form of the morning star Tlahuizcalpantecuhtli, which appears near the sun at sunrise. Quetzalcoatl’s journey signifies the continuous cycle of life.

Abstracted from http://www.mexconnect.com/mex_/travel/jcummings/diegomural3.html

